# Sizing the Depth and Width of Narrow Cracks in Real Parts by Laser-Spot Lock-In Thermography

**DOI:** 10.3390/ma14195644

**Published:** 2021-09-28

**Authors:** Mateu Colom, Javier Rodríguez-Aseguinolaza, Arantza Mendioroz, Agustín Salazar

**Affiliations:** Departamento de Física Aplicada, Escuela de Ingeniería de Bilbao, Universidad del País Vasco UPV/EHU, Plaza Ingeniero Torres Quevedo 1, 48013 Bilbao, Spain; mateu.colom@ehu.eus (M.C.); javier.rodriguezas@ehu.eus (J.R.-A.); arantza.mendioroz@ehu.eus (A.M.)

**Keywords:** laser-spot thermography, lock-in infrared thermography, nondestructive evaluation, crack detection

## Abstract

We present a complete characterization of the width and depth of a very narrow fatigue crack developed in an Al-alloy dog bone plate using laser-spot lock-in thermography. Unlike visible micrographs, which show many surface scratches, the thermographic image clearly identifies the presence of a single crack about 1.5 mm long. Once detected, we focus a modulated laser beam close to the crack and we record the temperature amplitude. By fitting the numerical model to the temperature profile across the crack, we obtain both the width and depth simultaneously, at the location of the laser spot. Repeating the process for different positions of the laser spot along the crack length, we obtain the distribution of the crack width and depth. We show that the crack has an almost constant depth (0.7 mm) and width (1.5 µm) along 0.7 mm and features a fast reduction in both quantities until the crack vanishes. The results prove the ability of laser-spot lock-in thermography to fully characterize quantitatively narrow cracks, even below 1 µm.

## 1. Introduction

Laser-spot thermography consists in heating the surface of an opaque sample with a focused laser beam and recording the surface temperature by means of an infrared (IR) video camera. In the absence of inner heterogeneities, the thermogram shows a symmetric distribution of the surface temperature around the laser spot. This symmetry is broken by the presence of subsurface defects (voids, inclusions, corrosion, delaminations, etc.). In the case of a vertical crack (typically, a fatigue crack) an abrupt temperature discontinuity along the length of the fissure appears in the thermogram, betraying the presence of the crack. By scanning the laser spot along the sample surface at constant velocity (the so-called flying spot thermography), large parts can be analyzed in a fast way [1,2]. This method is well suited to image cracks by showing the crack length along the surface [3,4,5,6,7,8,9,10,11,12,13,14,15,16,17]. A more challenging task is to evaluate the width and depth of the detected crack. By assuming that the vertical crack is infinite in depth, an analytical solution of the surface temperature can be obtained. In previous works, we have obtained the width of calibrated artificial cracks accurately by fitting this analytical solution to the experimental temperature data using either a modulated [18], a pulsed [19] or a continuously moving laser spot [20].

However, real cracks feature a finite penetration. If the depth of the crack is finite, the temperature distribution in the material cannot be written analytically and the solution has to be sought numerically. Several research groups have applied finite element methods (FEM) to solve the heat diffusion equation to simulate the surface temperature of a sample containing a finite crack when it is illuminated by a focused laser beam [21,22,23,24,25,26,27,28,29,30,31,32]. However, in these works, only cracks wider than 40 µm were studied because in classical continuous FEM, the cracked sample is divided into two domains: the bulk and the air filling the crack [33]. Accordingly, it is necessary to mesh the whole volume both outside and the inside the crack. Therefore, for very narrow cracks, this method requires very fine meshes, dramatically increasing memory resources and computing time. To overcome this limitation, in a previous work, we modeled the crack as an interface characterized by its thermal contact resistance. Accordingly, we were able to calculate the surface temperature of a sample containing a crack regardless of how narrow it was [34]. In order to check the validity of the procedure, we applied the methodology to narrow calibrated artificial cracks of constant width and depth, demonstrating that laser-spot lock-in thermography can be used to size simultaneously the width and depth of fissures by fitting the numerical model to the experimental temperature profile across the crack [34].

In this work, we present a complete and reliable local characterization of the width and depth of a very narrow natural crack developed in an Al-alloy dog bone plate with a drilled hole in the middle, which was subjected to successive cycles of compression and expansion. The photographic picture of the plate showed many scratches, but it was impossible to assess which of them, if any, was penetrating beneath the surface. The first task was to obtain a thermographic image of the part, which revealed the presence of a fissure about 1.5 mm long, starting at the notch. The reason for applying a modulated laser beam is that lock-in thermography is able to reduce the noise level in the data well below the noise equivalent temperature difference (NETD) of infrared cameras [35]. Then, we brought the laser spot close to the crack and recorded the temperature amplitude, which was fitted to the numerical model, thus obtaining the width and depth of the crack at the current position of the laser. We demonstrated that both quantities are not correlated, so they can be obtained simultaneously from the same amplitude thermogram. We repeated this procedure placing the laser spot at several positions covering the whole length of the crack. As a result, we obtained a complete characterization of the crack along the whole length: from the starting point at the notch until it vanishes.

## 2. Background

**Figure 1:**
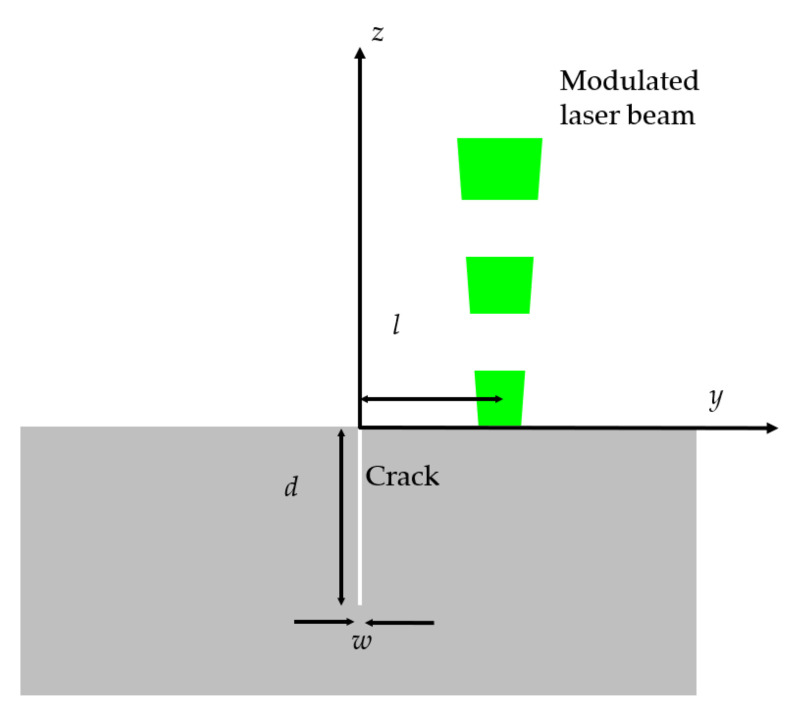
Cross-section of the sample with a vertical surface-breaking crack.

The most useful configuration to detect and characterize vertical cracks using laser-spot lock-in thermography is to focus a modulated laser beam close to the crack and record the surface temperature oscillation with an IR video camera. Figure 1 shows the geometry of the problem, where *l* is the distance between the center of the laser spot and the crack and *w* and *d* are the width and depth of the crack, respectively. The crack width is thermally characterized by the thermal contact resistance:

(1) $$R_{th}=\frac{w}{K_{air}}$$

where *K* is thermal conductivity [36]. The laser beam has a Gaussian profile; it is focused to a radius *a* (at 1/*e*^2^ of the maximum intensity), its power is *P_o_*, and its amplitude is modulated at a frequency *f* (*ω* = 2*πf*). The center of the laser spot is on the *y*-axis (0,*l*,0). The thermal diffusion length $µ=\sqrt{D/\left(\pi f\right)}$, where *D* is the thermal diffusivity of the sample, measures the distance traveled by the thermal wave before its amplitude almost vanishes (its amplitude is reduced by a factor 1/*e*). Accordingly, two cases can be considered: (a) if *µ* < *d*, the crack behaves as infinite in depth, and only the width of the crack can be retrieved; (b) if *µ* ≥ *d*, the crack acts as finite in depth, and therefore, both *d* and *w* can be obtained. As, for a given specimen, the thermal diffusion length depends on the modulation frequency, an appropriate selection of *f* allows the crack to be studied as infinite or finite.

### 2.1. Analytical Model: Vertical Cracks of Infinite Depth

If the depth of the crack is infinite (or if *d* >> *µ*) and adiabatic boundary conditions at the sample surface are assumed, an analytical expression for the surface temperature can be obtained. The temperature at both sides of the crack along the *y*-axis, crossing the crack through the center of the laser spot, is given by [18]

(2) $$T(0,y,0)=\frac{\eta P_{o}}{4\pi K}\int_{0}^{\infty }\delta J_{o}\left(\delta \left|y-l\right|\right)\frac{e^{-\frac{{\left(\delta a\right)}^{2}}{8}}}{\beta }d\delta +\;+sign(y)\frac{\eta P_{o}}{4\pi K}KR_{th}\int_{0}^{\infty }e^{-\frac{{\left(\delta a\right)}^{2}}{16}}I_{o}\left[\frac{{\left(\delta a\right)}^{2}}{16}\right]\frac{e^{\left(\frac{a^{2}\beta^{2}}{8}-\beta l-\beta \left|x\right|\right)}}{2+KR_{th}\beta }\left[1-erf\left(\frac{a^{2}\beta -4l}{2\sqrt{2}a}\right)-e^{2\beta l}erfc\left(\frac{a^{2}\beta +4l}{2\sqrt{2}a}\right)\right]\delta d\delta$$

Here, *η* is the laser power fraction absorbed by the sample, $\beta =\sqrt{\delta^{2}+{\left(\frac{i\omega }{D}\right)}^{2}}$; *J_o_* and *I_o_* are the Bessel function and the modified Bessel function of order zero, respectively; and *erf* and *erfc* are the error function and the complementary error function, respectively. *Sign*(*y*) is −1, 0, or 1 depending on whether *y* is negative, zero, or positive, respectively.

**Figure 2:**
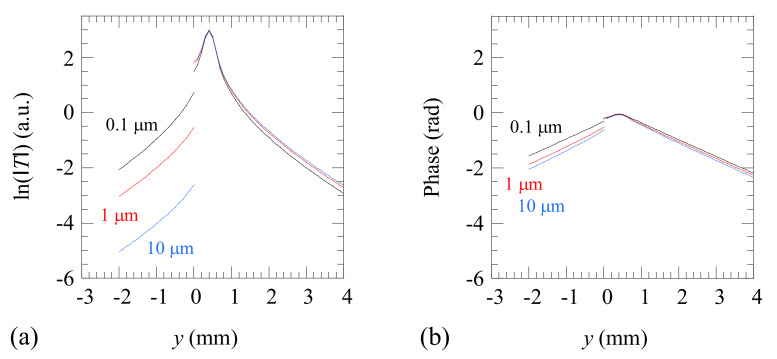
Calculations of (**a**) ln(|*T*|) and (**b**) phase along the *y*-axis for an Al-alloy sample (*D* = 42 mm^2^/s, *K* = 100 W m^−1^ K^−1^) containing an infinite vertical crack, located at *y* = 0. Three crack widths are considered: *w* = 0.1, 1, and 10 µm. The sample is illuminated by a Gaussian laser beam of power *P_o_* = 1 W modulated at *f* = 5 Hz. The laser spot has a radius of *a* = 0.2 mm, and it is located at a distance *l* = 0.4 mm from the crack.

In Figure 2 we show the calculations of the amplitude and phase of the temperature profile along the *y*-axis for an Al-alloy sample (*D* = 42 mm^2^/s, *K* = 100 W m^−1^ K^−1^), which is illuminated by a Gaussian laser beam of radius *a* = 0.2 mm, whose center is located at a distance *l* = 0.4 mm from the crack. The laser power is *P_o_* = 1 W and the modulation frequency is *f* = 5 Hz. Three crack widths are analyzed. The vertical axis represents the natural logarithm of the temperature in order to visualize small temperature differences that occur especially at the nonilluminated side, where the temperature is low. As can be observed, a discontinuity in both amplitude and phase appears at the crack position. This jump is due to the heat blockage produced by the fissure, and it increases as the width of the crack grows. Note that a crack as narrow as 0.1 µm produces a measurable temperature jump. This is due to the high thermal conductivity of the Al-alloy. Actually, Equation (2) has been written to show the correlation between the thermal resistance of the crack and the thermal conductivity of the sample through the product *KR_th_*. This means that it is easier to detect a narrow crack in a good thermal conductor than in a thermal insulator. Note that the same vertical scale has been used in amplitude and phase to clearly show that a much larger discontinuity is produced in amplitude than in phase and therefore the former is more appropriate to size the width of the crack. Accordingly, in the remainder of the paper, we focus only on temperature amplitude data.

The procedure to size the width of an infinite crack consists in fitting the experimental temperature profile perpendicular to the crack and crossing the center of the laser spot to Equation (2). As can be seen in Equation (2), there are four free parameters to be fitted: *a*, *l*, *ηP_o_*/*K*, and *KR_th_*. From the retrieved value of *KR_th_* and assuming that the thermal conductivity of the sample is known, we obtain the thermal contact resistance associated with the crack. Finally, from *R_th_*, the width of the crack *w* is obtained using Equation (1).

### 2.2. Numerical Model: Vertical Cracks of Finite Depth

Even if the analytical approach is useful in the modeling of infinite vertical cracks, the search for a more general modeling method is needed in order to include more realistic fissures. In these terms, numerical FEM provides a reliable alternative when more complex crack geometries are found. In particular, in this work, a numerical FEM model has been developed with the aim of analyzing vertical cracks with finite depth.

As it is well known, FEM provides a solution to the model equations over a spatial triangulation of the studied domain. Consequently, if the nature of the crack is fully determined, its geometrical implications can be introduced on the discretization in order to solve the heat diffusion equation, Equation (3), over the cracked domain. Additionally, the model boundary condition, Equation (4), is introduced as a heat flux due to the laser harmonic heating, $g(\vec{r},t)$, and convective/radiative surface heat losses modeled by means of an effective linear term $\gamma (T(\vec{r},t)-T_{amb})$. The initial temperature field is assumed to be constant and equal to ambient temperature (Equation (5)).

(3) $$\frac{\partial T\left(\vec{r},t\right)}{\partial t}-\nabla \cdot \left[\nabla D\left(\vec{r}\right)T\left(\vec{r},t\right)\right]=0$$

(4) $$K(\vec{r})\nabla T(\vec{r},t)\cdot \hat{n}{|}_{S}=g(\vec{r},t)-\gamma \left[T(\vec{r},t)-T_{amb}\right]$$

(5) $$T(\vec{r},t_{0})=T_{amb}$$

where $\hat{n}{|}_{S}$ represents the normal direction to the sample surface and γ is the effective convective/radiative heat loss coefficient.

Since lock-in thermography uses a harmonic heat source, the heat supply $g(\vec{r},t)$ can be modeled as the addition of a stationary and a periodic contribution:

(6) $$g(\vec{r},t)=g(\vec{r})\left[1+\cos(\omega t)\right]$$

where $g(\vec{r})=\frac{\eta P_{o}}{\pi a^{2}}e^{-\frac{2r^{2}}{a^{2}}}$ stands for the half of the maximum power supplied by the excitation laser source with spatial Gaussian distribution. Accordingly, once the steady state has been reached, the temperature field can be modeled as the addition of the initial temperature, *T_amb_*; the stationary temperature rise, $T_{dc}(\vec{r})$; and the harmonic contribution, $T_{ac}(\vec{r},t)$, which is usually called thermal wave:

(7) $$T(\vec{r},t)=T_{amb}+T_{dc}(\vec{r})+T_{ac}(\vec{r})\cos\left(\omega t\right)$$

Since we are only interested in computing the harmonically oscillating temperature, it is enough to remove the constant in Equation (7) leading to $T(\vec{r},t)=T_{ac}(\vec{r})\cos\left(\omega t\right)$, i.e., the thermal wave.

**Figure 3:**
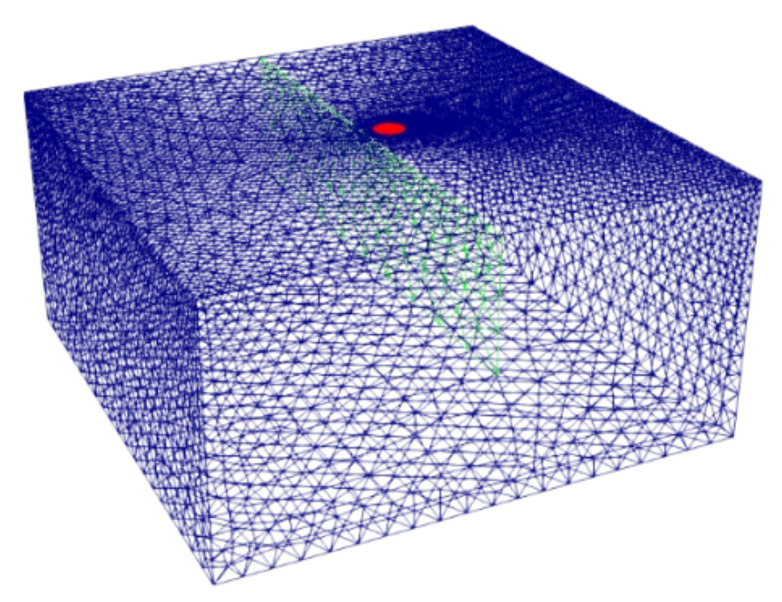
Triangulation of the investigated sample domain. The crack is depicted in green as a 2-D interface, whereas the laser heat supply is depicted as a red circle.

Regarding the crack, it is introduced in the domain as a two-dimensional interface with a given thermal resistance, calculated by Equation (1). This crack modeling strategy presents important advantages when compared to the conventional introduction of the crack as a finite volume filled with air, including the following:

(1) The crack is introduced in a natural manner as a 2-D conformal interface on the domain triangulation (see Figure 3) leading to the low-complexity spatial discretizations.
(2) The crack internal domain (air) is not required to be triangulated since it is modeled as a thermal resistance boundary (see Figure 3).
(3) Very efficient computation, avoiding calculations over very large or complicated meshes.

Considering that the crack is introduced as a boundary interface, the heat flux through the crack and the temperature difference between both sides of the crack satisfy the following equations:

(8) $$\left[\left[K\nabla T\left(\vec{r},t\right)\right]\right]{|}_{Crack}=0$$

(9) $$\Delta T\left(\vec{r},t\right){|}_{Crack}=R_{th}K\nabla T\left(\vec{r},t\right){|}_{Crack}$$

where the [[ ]] operator stands for “jump” on the heat flux over the crack grid nodes.

Overall, Equations (3)–(5) together with Equations (8) and (9) form a complete set of equations, which provide a solution for the thermal wave temperature, $T(\vec{r},t)=T_{ac}(\vec{r})\cos\left(\omega t\right)$, obtained by the thermal solver of the OpenFOAM package [37]. Once the thermal wave is obtained (direct problem), its spatially dependent amplitude, |*T*|, is programmatically computed. Finally, a constrained gradient-based method for nonlinear multiparametric optimization has been developed in order to fit the theoretical results to experimental data (inverse problem). As a result, the investigated narrow crack properties (width and depth) are retrieved.

For reproducibility reasons, the practical information regarding the mesh size and computation of both the direct and inverse problems described above are summarized in Table 1.

**Figure 4:**
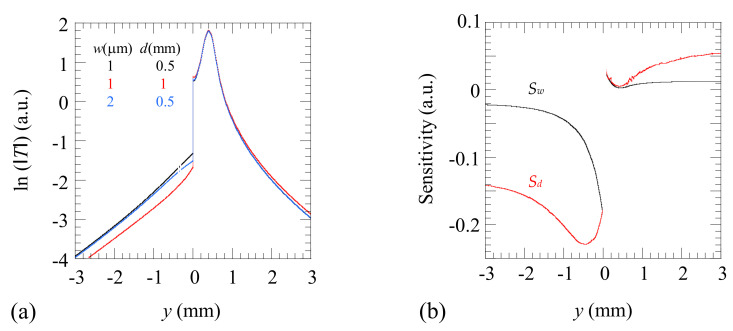
(**a**) Simulation of ln(|*T*|) as a function of the transverse distance to the crack, which is located at *y* = 0. Simulations have been performed for an Al-alloy sample (*D* = 42 mm^2^/s, *K* = 100 W m^−1^ K^−1^) with the same experimental parameters as in Figure 2: *P_o_* = 1 W, *f* = 5 Hz, *a* = 0.2 mm, and *l* = 0.4 mm. Three couples of (*w*, *d*) are plotted. (**b**) Simulation of the sensitivity of ln(|*T*|) to *w* and *d* for the same parameters.

In order to illustrate the effect of a crack of finite penetration on the surface temperature, in Figure 4a we show simulations of ln(|*T*|) along the *y*-axis obtained using the above FEM model applied to an Al-alloy (*D* = 42 mm^2^/s, *K* = 100 W m^−1^ K^−1^) plate containing a finite-depth crack. The same experimental parameters (*P_o_* = 1 W, *f* = 5 Hz, *a* = 0.2 mm, and *l* = 0.4 mm) as in Figure 2 have been used in the simulations. Three couples (*w*, *d*) are analyzed. The black line is the simulation for a 1 µm wide crack penetrating 0.5 mm beneath the surface. We focus our analysis on narrow cracks because we are interested in the early detection of fatigue cracks, such as initial cracks developed in Al-alloy parts subjected to cycles of tensile and compressive forces. The calculated profiles feature significantly large discontinuities at the position of the crack, as expected for such a good thermal conductor. Then, in order to analyze the impact of variations of both parameters in ln(|*T*|), first we double the depth while keeping the width fixed (1 µm, 1 mm): the red curve. As can be observed, doubling the depth produces a displacement of ln(|*T*|) downwards at the nonilluminated side of the crack, almost parallel to the black curve. Then we come back to the initial depth while doubling the width of the crack (2 µm, 0.5 mm): the blue line. Note that doubling the width of the crack only produces a small deviation downwards of the curve at the nonilluminated side, close to the crack, whereas further away the blue and black lines remain almost indistinguishable. These results indicate that ln(|*T*|) is more sensitive to *d* than to *w* for narrow cracks. Accordingly, it is expected that the depth will be quantified with higher precision than the width.

There is a remaining question of whether both magnitudes, *w* and *d*, are correlated, i.e., whether the same temperature profile can be reproduced by several couples (*w*, *d*). In order to clarify this point, we have calculated the sensitivity of ln(|*T*|) to *w* and *d* according to the following definition:

(10) $$S_{p}=p\frac{\partial \ln(\left|T\right|)}{\partial p}\;\;\mathrm{with}\;p=w,d$$

In Figure 4b, we plot the sensitivity of the *y*-profile to both parameters in the case of a crack with *w* = 1 µm and *d* = 0.5 mm. The sensitivity to the width (*S_w_*) is shown in black, and the sensitivity to the depth (*S_d_*) is shown in red. As can be observed, the sensitivity to both parameters is very low at the illuminated side of the crack, but it is noticeable at the nonilluminated side. On the other hand, it can be observed that both quantities are not correlated (their sensitivities are not proportional), and therefore they can be obtained univocally from one temperature profile. Moreover, it is confirmed that, as it was already indicated in Figure 4a, $\left|S_{d}\right|>\left|S_{w}\right|$.

In order to obtain both the width and depth of a finite-depth crack, the experimental temperature profile perpendicular to the crack and crossing the center of the laser spot is used as an input for the developed aforementioned multiparametric inverse model. In this case, there are five free parameters (*a*, *l*, *ηP_o_*/*K*, *KR_th_*, and *d*) to compute, from which the desired crack properties are obtained: *w* and *d*.

## 3. Experimental Results and Discussion

**Figure 5:**
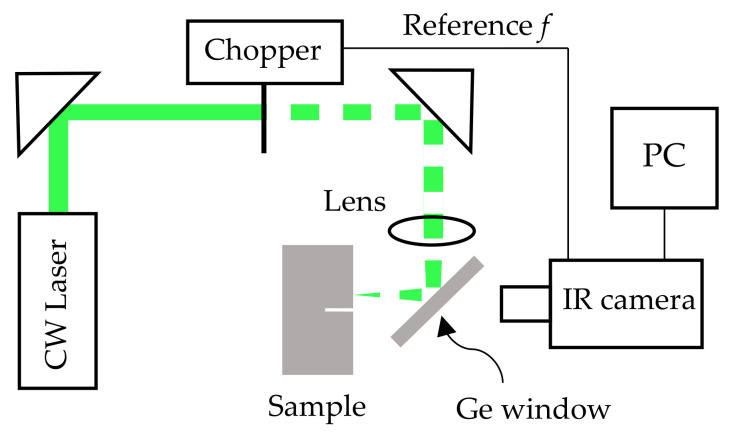
Scheme of the laser-spot lock-in thermography setup.

In Figure 5, we show a scheme of the laser-spot lock-in thermography setup. The sample is excited by means of a CW laser (Coherent Verdi, 532 nm, up to 6 W) of Gaussian profile. The beam intensity is modulated by means of a mechanical chopper and is focused down to a radius of about 200 µm on the sample surface using a 10 cm focal length lens. The beam is directed perpendicularly to the sample surface by reflection on a Ge window, which is transparent to the IR radiation emitted by the sample. The camera (FLIR SC 7500, 3–5 µm, 256 × 320 px, NETD < 20 mK, 30 µm pitch and up to 380 images/s at full frame), synchronized with a mechanical chopper, detects and displays the evolution of the surface temperature for several seconds. Once the steady state has been reached, a lock-in module incorporated into the camera analyzes the recorded film at the modulation frequency and extracts the amplitude of the temperature oscillations at each pixel together with the phase lag with respect to the excitation signal (coming from the chopper), delivering an amplitude thermogram and a phase thermogram. A macro lens in the camera produces a magnification ratio of 1:1; i.e., each pixel of the detector senses the average temperature over a 30 µm square of the sample. The sample is mounted on a micropositioning system to control the position where the laser spot excites the surface and the distance to the fissure, with an accuracy of 10 µm.

**Figure 6:**
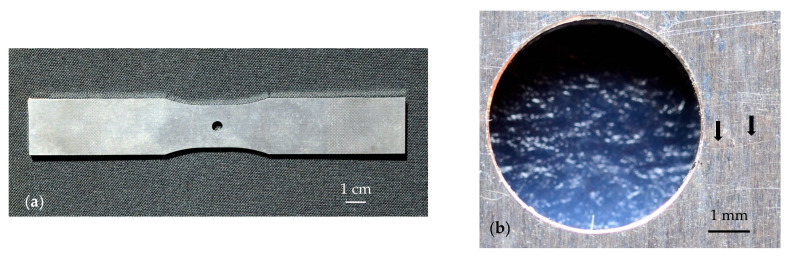
(**a**) Photograph of the dog bone Al-alloy plate. (**b**) Close-up picture of the sample around the hole. Arrows indicate the position of the crack, which was identified after obtaining its thermographic image.

The sample we are dealing with is a dog bone plate 2 mm thick with lateral dimensions 17 cm and 2.8 cm made of Al-alloy 2034, whose thermal properties are the same as those used in Figure 2 and Figure 4: *D* = 42 mm^2^/s, *K* = 100 W m^−1^ K^−1^. In the middle of the sample, a 5 mm diameter hole was drilled (see Figure 6a). This plate was subjected to successive compression and tension cycles to test its mechanical resistance to fatigue, and the thermographic experiments were conducted after removing the part from the load machine. It is expected that some fissures are produced around the hole during this process. A close-up photograph of the sample is shown in Figure 6b. As can be seen, there are many lines at the surface, and it is difficult to distinguish whether they are harmless surface scratches or harmful surface-breaking cracks. Accordingly, the first goal is to assess which of those lines are cracks, and the second, more challenging, one is to size their width and depth. We deposited a very thin graphite layer (a few microns thick) at the illuminated surface to enhance both the laser light absorption and the IR emissivity.

**Figure 7:**
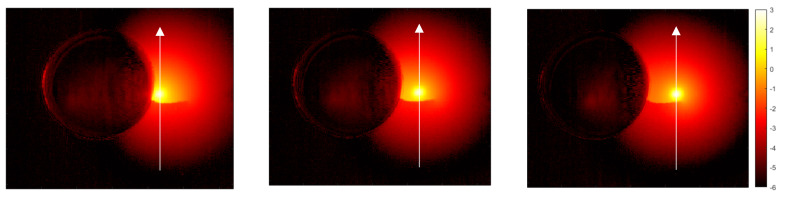
Natural logarithm amplitude thermogram at three different positions of the laser spot at *f* = 11 Hz. The temperature discontinuity produced by the crack is clearly noticeable. The white arrows indicate the profiles that will be analyzed for sizing the crack.

**Figure 8:**
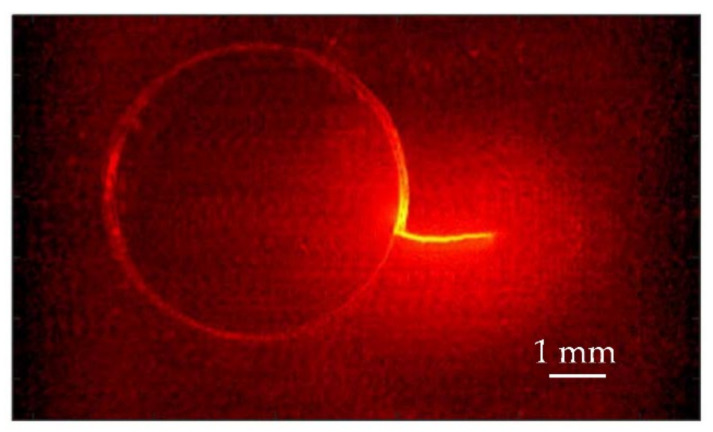
Combined first-derivative thermographic image of the crack.

In order to detect the crack and to obtain a qualitative image of it, we follow the method proposed by Almond and coworkers in 2011 [9,10]. First, we obtain several temperature amplitude thermograms at different positions of the laser spot on the sample surface, obtained by displacing the sample by distances multiples of the pixel size (30 µm) by means of the micropositioning system. In all of them, the modulation frequency is kept fixed. In Figure 7 we show the amplitude thermograms at three positions of the laser spot obtained at *f* = 11 Hz. We plot the ln(|*T*|) rather than the temperature itself to expand the low temperature values. Then, in order to identify eventual temperature discontinuities associated with cracks, we compute the first spatial derivative along the *x*-axis and along the *y*-axis of each amplitude thermogram. This is simply done by calculating temperature differences of adjacent pixels. At that moment, for each position of the laser, we generate a new image by adding the squares of the first-derivative images in the *x* and *y* directions to show the derivative information in both directions simultaneously. Finally, we combine all the first-derivative images in a unique derivative thermogram (see Figure 8). This requires appropriately shifting the derivative images obtained for different positions of the sample. It can be observed that, apart from the hole itself, only one of the surface lines is confirmed as a flaw: the one that is marked by arrows in the optical picture in Figure 6b. Therefore, the other lines are mere surface scratches with no depth penetration.

To size the depth and width of the confirmed crack, we focus the laser spot close to the crack (≈0.4 mm) at seven positions covering the whole length of the fissure. For each position, we record the temperature amplitude thermogram, from which we extract the vertical profile crossing the center of the laser spot (see the white arrows in Figure 7).

**Figure 9:**
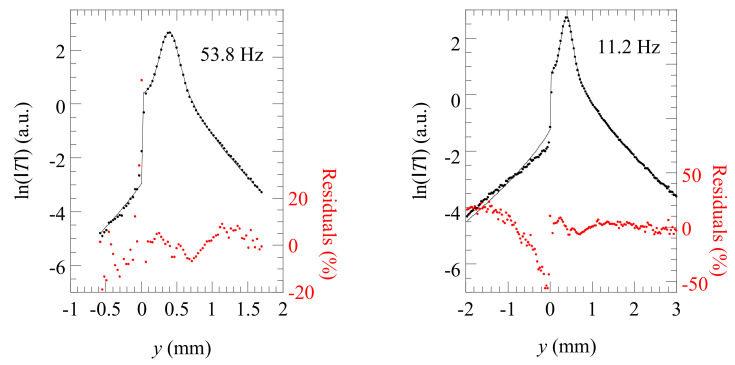
**E**xperimental ln(|*T*|) profiles corresponding to the left thermogram in Figure 7 for two modulation frequencies. Dots are the experimental results, and the continuous line is the fitting to Equation (2). Residuals are plotted to visualize the quality of the fit.

Figure 9 shows by dots the temperature amplitude profile corresponding to the first position in Figure 7 for two modulation frequencies: 53.8 and 11.2 Hz. As a first trial, we fitted those profiles to Equation (2), which assumes that the crack is infinite in depth. The results of the fittings are the continuous lines in Figure 9. From the retrieved value of *R_th_*, we obtain the (air gap) width of the crack *w* using Equation (1). Additionally, we check the consistency of the results by comparing the fitted values of *a* and *l* with the optically measured laser radius and distance to the crack (*a* ≈ 200 µm and *l* ≈ 400 µm). In Figure 9, we also plot the residuals (the difference between experimental and fitted temperature values) to visualize the quality of the fits. As can be observed, the quality of the fit is very good for 53.8 Hz, with statistical noise of about 10%, leading to a crack width *w* = 1.5 ± 0.4 µm. However, the model clearly fails at 11.2 Hz, since it is not able to fit the temperature at the nonilluminated side of the crack. This behavior can be understood by analyzing the thermal diffusion length for each frequency: 0.5 mm at the higher frequency against 1.1 mm at the lower frequency. At 53.8 Hz, the fitting to Equation (2) is very good, indicating that the actual depth of the crack is greater than 0.5 mm, and thus the crack behaves as infinitely deep at this frequency. On the contrary, the bad fitting at 11.2 Hz indicates that the depth of the crack verifies *d* ≤ 1.1 mm, and the infinite crack model does not describe the effect of the crack on the surface temperature.

**Figure 10:**
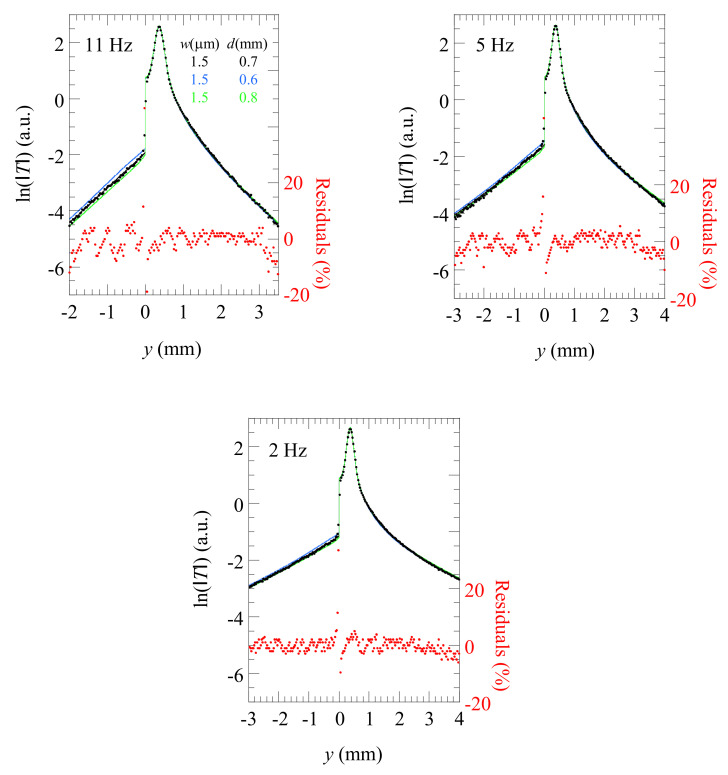
Experimental ln(|*T*|) profile corresponding to position 1 in Figure 7 for low modulation frequencies. Dots are the experimental results, and the continuous black line is the fitting to the developed FEM model. The vertical scale is kept fixed for a better comparison of the results. Residuals are plotted to visualize the quality of the fit.

Now, in order to obtain the width and depth of the crack, the obtained experimental data are used as input for the developed aforementioned multiparametric inverse model. According to the results of the previous paragraph, we avoid too high frequencies for which the thermal diffusion length is smaller than the crack depth. In Figure 10, we show by dots the temperature profiles corresponding to the first thermogram in Figure 7 at three modulation frequencies fulfilling the mentioned criterion: 11, 5, and 2 Hz, for which the thermal diffusion lengths are 1.1, 1.6, and 2.6 mm, respectively. The same vertical scale of the temperature has been used for a better comparison of the results. The black lines are the best fittings to the numerical model. The residuals are also plotted to assess the good quality of the fittings, and they prove that the numerical model describes the experimental data very accurately. For the three frequencies, we obtained the same values of the parameters: *w* = 1.5 ± 0.3 µm and *d* = 0.7 ± 0.05 mm, regardless of the initial guesses of the inverse multiparametric fitting model. Note that, as predicted by the sensitivity analysis, the uncertainty in depth (≈7%) is smaller than the uncertainty in width (≈20%). To better appreciate the resolution of the method, for each frequency, we have plotted the temperature profiles for two depths covering the range of the uncertainty: *d* = 0.8 (blue line) and 0.6 mm (red line). As can be observed, although we do not have an independent estimation of the crack depth, it is obvious that the curves corresponding to depths of 0.6 and 0.8 mm do not match the experimental data. However, it has to be noted that, when reducing the modulation frequency, the signal-to-noise ratio is increased but the sensitivity is reduced. A wise procedure to measure *w* and *d* is to avoid high frequencies, for which the crack behaves as infinitely deep, and also too low frequencies, for which *µ* >> *d*, since the resolution is reduced. Actually, the best tradeoff is to select a frequency verifying *µ* ≈ *d*.

It is worth mentioning that the width of the crack remains constant along its whole depth in our model (see Figure 1). However, it is expected that the width decreases with depth in real cracks. Accordingly, the width value *w* that we obtain from the fitting to the model is an effective width.

**Figure 11:**
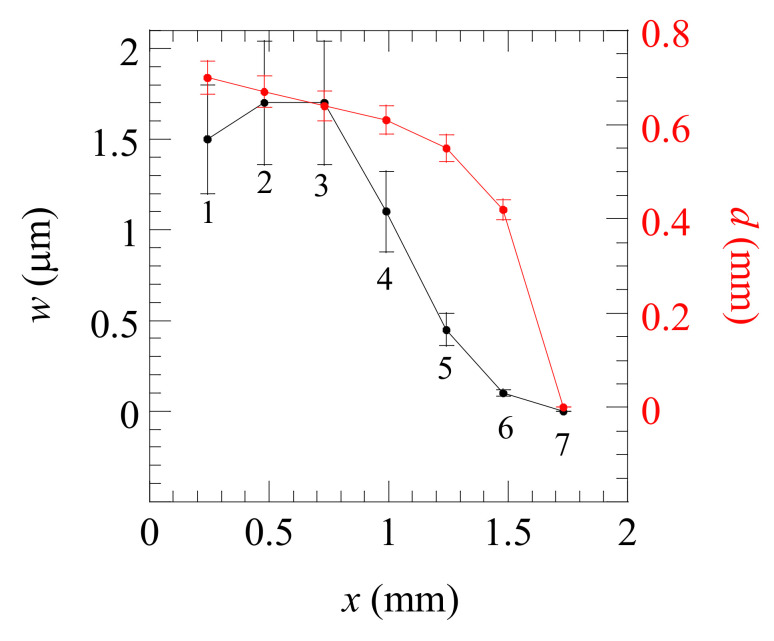
Fitted values of the width and depth of the crack for each of the seven positions marked in Figure 7. *x* corresponds to the distance to the hole. The lines are a guide for the eye.

We have performed the same analysis for seven laser positions covering the whole crack length. The results are summarized in Figure 11, indicating that for the first 0.7 mm, the depth and width of the crack remain almost constant, and then both are reduced until the fissure vanishes. This information is interesting for industrial partners to assess the condition of the part. Moreover, this approach offers the possibility of analyzing the evolution of the crack as it grows by repeating this IR thermography study as the number of cycles of compression and expansion is increased. To the best of our knowledge, this is the first time that the distribution of the width and depth of a crack in the micrometer range is obtained along the whole length of the fissure. It has to be noted that the minimum crack width that can be detected and sized using this technique depends on the thermal properties of the material according to the *KR_th_* dependence of the temperature field. Accordingly, the minimum crack width detectable in a thermal insulator will be wider than in this Al-alloy.

## 4. Conclusions

Conventional NDT methods such as dye penetrants are efficient in detecting wide cracks. However, imaging and sizing narrow cracks is a challenge, as they can be confused with mere surface scratches. Some nondestructive testing techniques such as ultrasounds are able to detect very narrow cracks (wider than the displacement fields, about 5 nm), although determining the crack depth might be problematic. In this work, we have shown that laser-spot lock-in thermography features a high enough sensitivity to image very narrow early-stage fatigue cracks and distinguish between true cracks and mere superficial scratches. Most importantly, we have experimentally demonstrated that by combining numerical calculations using finite elements and laser-spot lock-in thermography it is possible to determine the local values of the crack width and depth at different positions along the crack length, for cracks as narrow as 1 µm in Al-alloy. The simulations show that even 0.1 µm wide cracks can be analyzed in this material. However, because the temperature discontinuity that characterizes the crack depends on the product *KR_th_*, the minimum width of the cracks that can be detected is narrower in good conductors.

As the technique enables full local characterization of the length, width, and depth of the crack, this methodology provides an opportunity to analyze the dynamics of the crack growth in fatigue tests by performing the study at several stages during the test.

## Figures and Tables

**Table 1:** Finite element model mesh and hardware information. Distances expressed as multiples of the thermal diffusion length *µ*.

Mesh overall size (spatial directions as in Figure 1)	*x*-direction: 14*µ* *y*-direction: 14*µ* *z*-direction: 7*µ*
Mesh cell number	75,000 (average)
Hardware	Intel Xeon Gold 5218 2.3–3.9 GHz 16 core/32 thread RAM DDR4 128 Gb
Direct problem computing time	3 min (average)
Inverse problem computing time	200 min (average)
